# The Sternalis Muscle—Incidental Finding of a Rare Chest Wall Muscle Variant During Keloid Excision-Chest Wall Reconstruction

**Published:** 2012-08-10

**Authors:** Dinh T. Nguyen, Rei Ogawa

**Affiliations:** ^a^Department of Plastic, Reconstructive and Aesthetic Surgery, Nippon Medical School Hospital, Tokyo, Japan

## Abstract

**Introduction:** The sternalis is a rare (strap-like) parasternal muscle that is well known to anatomists, but relatively unknown to clinicians, including surgeons. Familiarity with the muscle is important in avoiding potential confusion when incidentally encountered. When available, the muscle can be harvested for reconstruction of the chest wall or of nearby region. **Presentation of Case:** The patient is a 55-year-old man with significant sternal keloids secondary to a previous history of severe acne. The patient desires removal of the keloids. Hence, a decision was made to excise the keloids, followed by immediate reconstruction with a propeller flap. Intraoperative excision of the keloids and undermining of adjacent subcutaneous tissue revealed chest muscle fibers fitting the description of the sternalis muscle. The patient tolerated the procedure without any complications. **Discussion:** The sternalis muscle can be confused for a mass on mammography, but confusion is resolved by computed tomography/magnetic resonance imaging. It has unclear embryonic origin—perhaps a remnant of the panniculus carnosus and/or derivative of a primitive ventral-longitudinal muscle sheet that give rise to the sternocleidomastoid and the rectus abdominis muscles. It is uni- or bilateral and has 1 or 2 bellies. It originates in the intraclavicular region and inserts onto the rectus sheath, costal cartilages, lower ribs, or external oblique aponeurosis. **Conclusion:** Not enough is known about the sternalis muscle to draw any conclusion about its utility in reconstructive surgery. It is hoped that cases will be presented in the foreseeable future describing its usage in reconstruction of the neck, chest, abdomen, and perhaps even other places.

The sternalis muscle is a rare (strap-like) anterior chest wall muscle variant with fibers near-parallel to the sternum and perpendicular to the pectoris major. Though inadequately described in standard anatomical texts, this muscle is familiar to trained anatomists.^1^ However, it is relatively unknown to radiologists, surgeons, and other clinicians until recently.^2^ Its rarity (roughly 5%-10% of the population),^3^ variable morphology,^4^ and poorly understood embryology^1^ render the muscle rather perplexing to anatomists, radiologists, and surgeons alike.

First presented in 1605 by Cabrolius,^5^ insights into the nature of the sternalis muscle have been ascertained mostly through cadaveric dissections.^4^ The recent use of mammography for breast cancer screening has increased awareness of this muscle (can be mistaken for a mass on mammography, but resolved by computed tomography/magnetic resonance imaging).^6^ There are few reported cases of involving the sternalis muscle in noncadavers; known cases are associated with breast cancer surgery.^7^^-^9 In this report, we present a case of incidental, intraoperative finding of the sternalis muscle during a routine keloid excision with immediate reconstruction in a male patient.

## CASE REPORT

**Figure 1:**
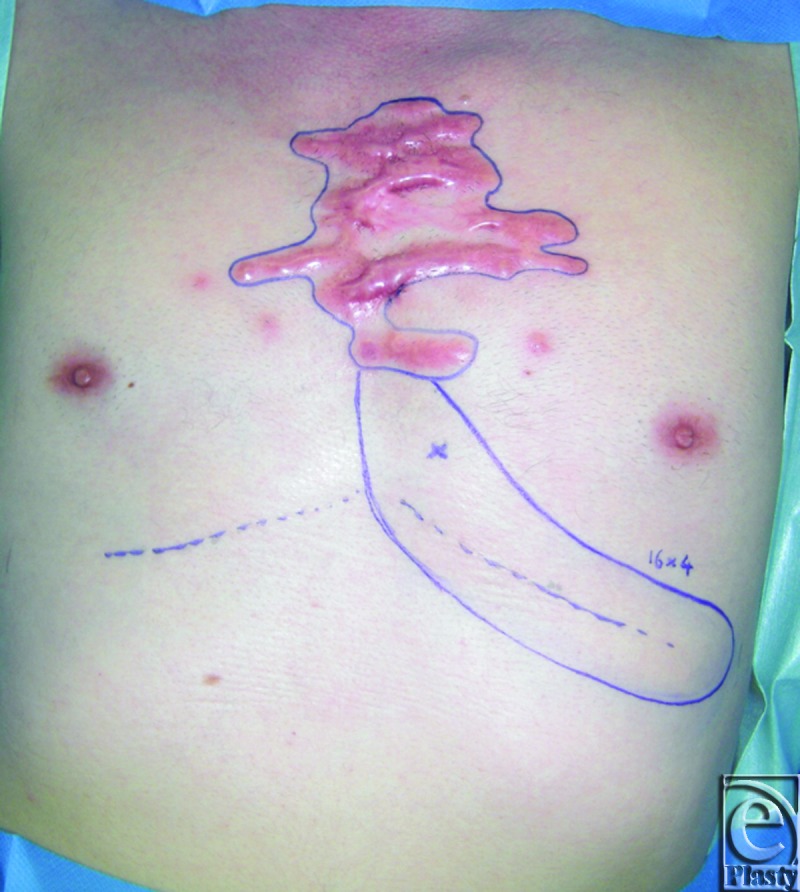
Design of the flap.

**Figure 2:**
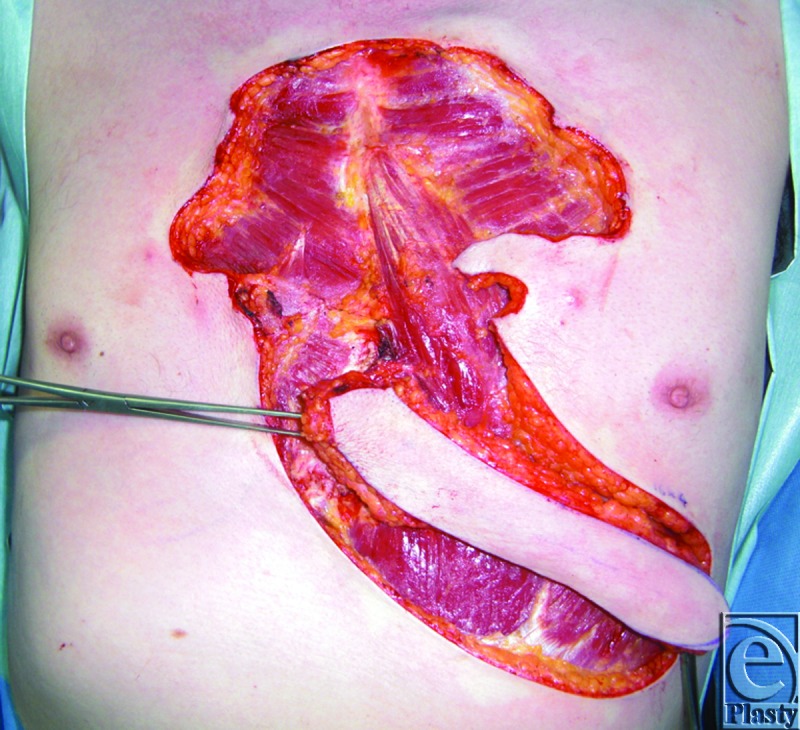
Elevation of the propeller flap.

**Figure 3:**
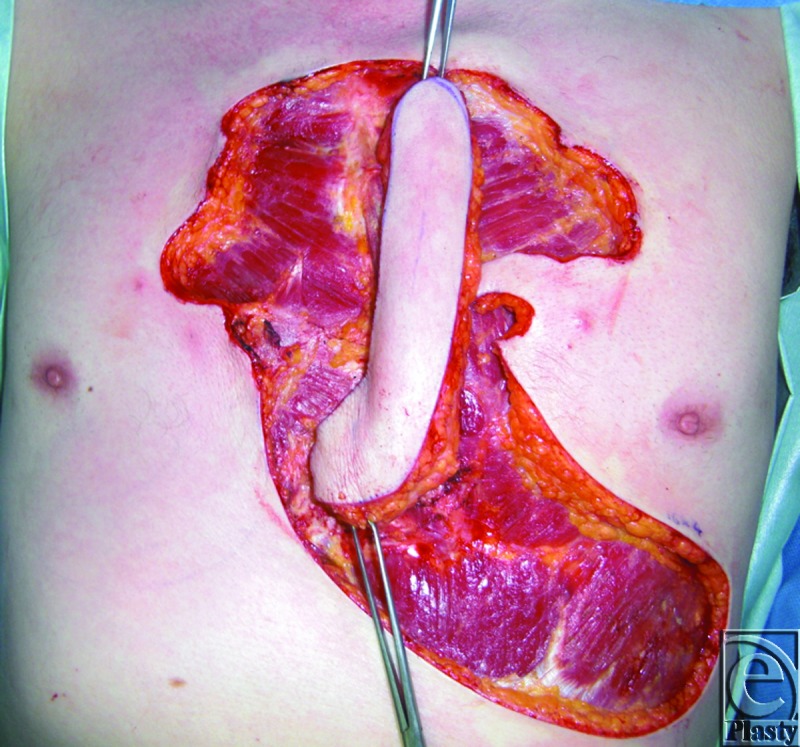
Rotation of the propeller flap.

The patient is a 55-year-old man with significant sternal keloids secondary to a previous history of severe acne. The patient desires removal of the keloids. Hence, a decision was made to excise the keloids, followed by immediate reconstruction with a propeller flap (Figs 1—3).

**Figure 4:**
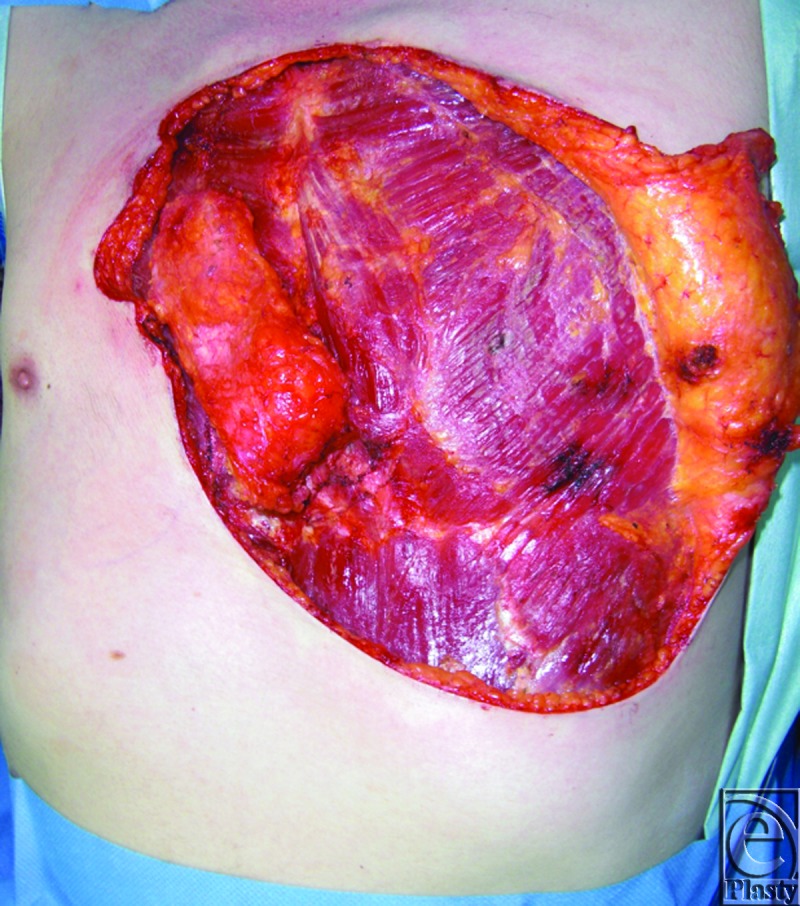
The sternalis muscle.

Intraoperative excision of the keloids and undermining of adjacent subcutaneous tissue revealed chest muscle fibers fitting the description of the sternalis muscle (Fig 4). The left parasternal muscle measures about 5 to 6 cm and appears to originate from the intraclavicular region (further dissection was not indicated) with insertion in the region where the left abdominal rectus sheath, left external oblique aponeurosis, and lower ribs margin converge. Tendon at the origin appears to be contiguous with tendon of the sternocleidomastoid muscle.

**Figure 5:**
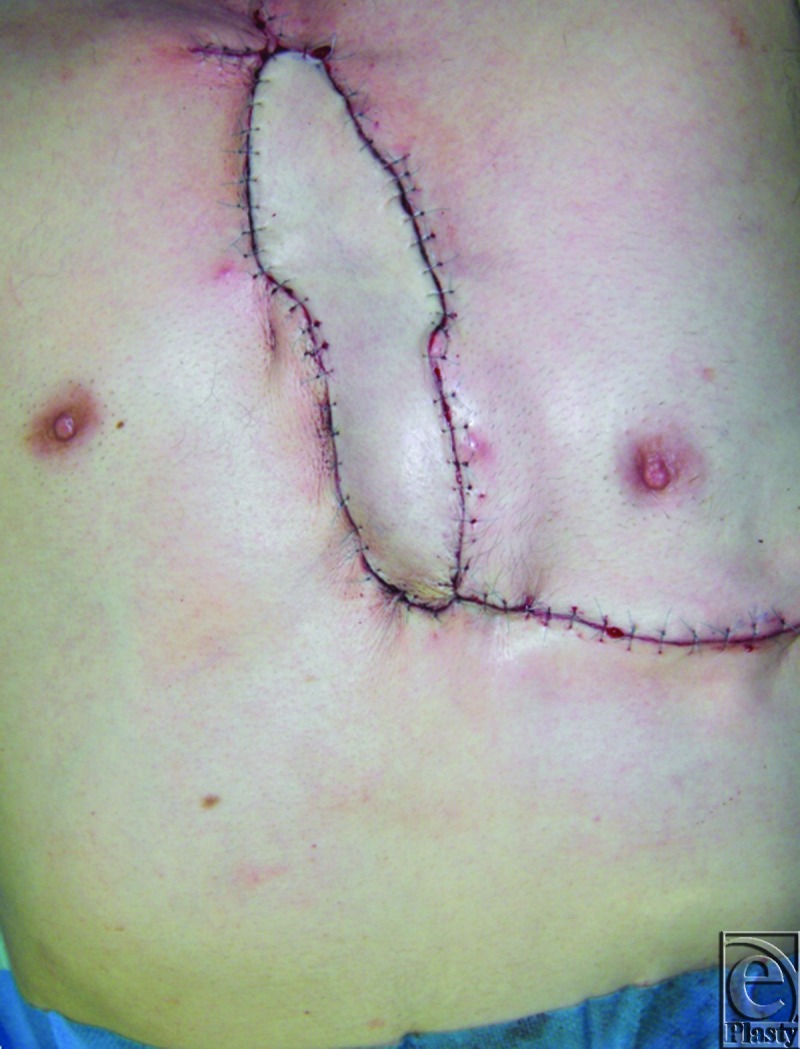
Immediately after operation.

**Figure 6:**
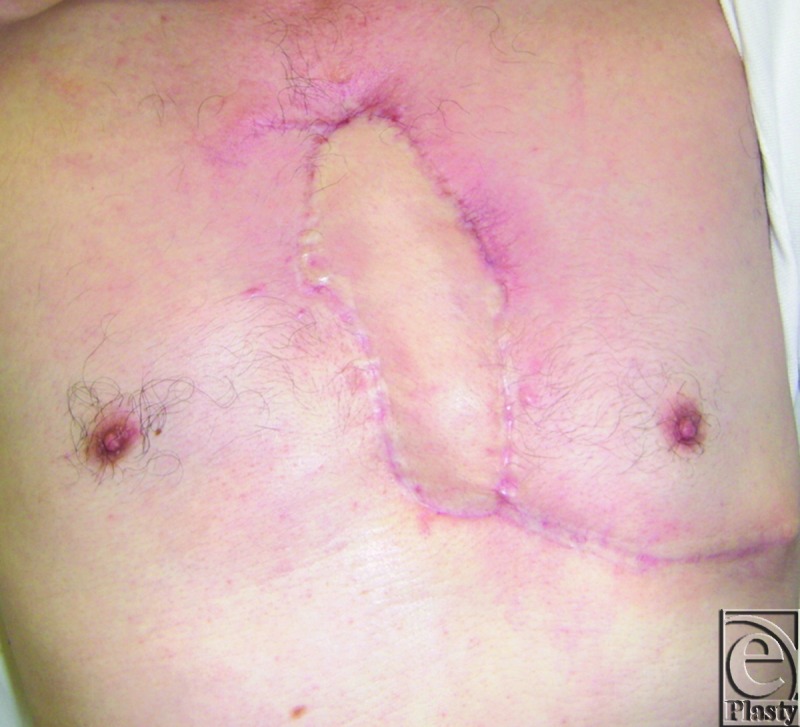
One year after operation.

The patient tolerated the procedure without any complications (Fig 5). He also underwent postoperative therapeutic radiation to help prevent keloid recurrence. Peripostoperative follow-up visit revealed a well-healed scar with mile erythema along the wound-edge union (Fig 6). There were no signs or symptoms of infection.

## DISCUSSION

To summarize, the sternalis muscle is a rare, superficial chest wall muscle that is inadequately described in standard anatomical texts. Although well known to trained anatomists, the muscle is relatively unknown to surgeons and other clinicians.^2^ The advent of radiographic tools has brought renewed interest in the muscle outside anatomical communities. More recently, intraoperative encounter of the muscle has been described^7^^-^9 and its utilization (when available) for chest wall reconstruction has been suggested.^7^^,^^10^^,^^11^ Only 1 case, in which the sternalis is employed for reconstruction (of the breast, in conjunction with a tissue expander), has been reported.^12^ Therefore, familiarity of the sternalis muscle not only broadens the surgeon's knowledge of variations of chest wall anatomy but also provides reconstructive options (when present) for wounds in the chest wall as well as adjacent regions.

As aforementioned, the sternalis is a parasternal, strap-like muscle with variable morphology and unclear embryonic origin. According to Jelev et al,^4^ the sternalis (1) lies between the anterior thoracic superficial fascia and the pectoral fascia, (2) originates in the intraclavicular region or sternum, and (3) inserts onto the rectus sheath, costal cartilages, lower ribs, or external oblique aponeurosis. The muscle is innervated by branches of the internal/external thoracic nerve and/or branches of the intercostal nerves. The muscle's function (help elevate the lower chest wall) is insignificant; therefore, it can be sacrificed for reconstruction. The internal thoracic artery provides blood supply to the muscle.

The sternalis muscle can be uni- or bilateral and may have 1 or 2 bellies.^4^ In instances when the muscle has 2 bellies, the major belly is always parasternal, whereas the minor belly can be found either parallel or orthogonal to the major belly (with bifurcation about the origin). It important to note that, although the belly and the origin are typically ipsilateral, contralateral orientation is also found. Presence of the sternalis is associated with partial absence of the ipsilateral (subjacent) pectoralis muscle.

Currently, there is great debate surrounding the embryologic origin of the sternalis muscle.^1^^,^^13^ This, in part, is due to variations of muscle morphology in postnatal encounters. It is thought that the sternalis may represent remnants of the panniculus carnosus.^5^^,^^14^ However, the muscle may also be a derivative of a primitive ventral-longitudinal muscle sheet that gives rise to the sternocleidomastoid and the rectus abdominis muscles.^15^ This would explain why tendon/fascial fibers of the sternalis are oftentimes contiguous with those of the sternocleidomastoid muscle and/or the rectus abdominis muscle. In even rarer cases, where the minor belly is perpendicular to the major belly, incomplete fusion with the pectoralis during prenatal development may be the explanation.^1^ Still, it is entirely possible that the muscle is derived from multiple sources.^16^

Irrespective of how the sternalis muscle is derived, it is important to be acquainted with its existence in order to avoid potential confusion when incidentally encountered. Moreover, when its presence is radiographically confirmed, in the setting of reconstructive potential (such as existing regional soft tissue defect), surgeons may elect to harvest the muscle for reconstructive use; sacrificing such “insignificant” muscle is always preferred over harvesting part of a more important functional muscle.

Not enough is known about the sternalis muscle to draw any conclusion about its utility in reconstructive surgery. It is hoped that cases will be presented in the foreseeable future describing its usage in reconstruction of the neck, chest, abdomen, and perhaps even other places.
